# Evidence of miR‐153‐3p association with Alzheimer's disease (AD) and the mechanism of miR‐153‐3p on critical proteins suggest a therapeutic and biomarker potential of miR‐153‐3p in AD and related dementias

**DOI:** 10.1002/alz70862_110012

**Published:** 2025-12-23

**Authors:** Debomoy K. Lahiri, Ruizhi Wang, Bryan Maloney, Bernardino Ghetti, Kwangsik Nho, Martin R. Farlow, Andrew J. Saykin, Fletcher A White, Kumar Sambamurti, Scott E. Counts

**Affiliations:** ^1^ Indiana Alzheimer's Disease Research Center, Indianapolis, IN USA; ^2^ Indiana University School of Medicine, Department of Psychiatry, Indianapolis, IN USA; ^3^ Indiana University School of Medicine, Department of Pathology & Laboratory Medicine, Indianapolis, IN USA; ^4^ Department of Radiology and Imaging Sciences, Indiana Alzheimer’s Disease Research Center, Center for Neuroimaging, Indiana University School of Medicine, Indianapolis, IN USA; ^5^ Department of Neurology, Indiana University School of Medicine, Indianapolis, IN USA; ^6^ Indiana Alzheimer's Disease Research Center, Indiana University School of Medicine, Indianapolis, IN USA; ^7^ Department of Radiology and Imaging Sciences, Indiana University School of Medicine, Indianapolis, IN USA; ^8^ Indiana University School of Medicine, Department of Anesthesia, Indianapolis, IN USA; ^9^ Medical University of South Carolina, Department of Neurosciences, Charleston, SC USA; ^10^ Michigan Alzheimer’s Disease Research Center, Ann Arbor, MI USA; ^11^ Michigan State University, Grand Rapids, MI USA

## Abstract

**Background:**

The most common neurodegenerative disorders include Alzheimer's disease (AD), Lewy body and related dementias (ADRDs). Triggers of pathobiochemical changes in ADRDs remains unknown and appear numerous. Short non‐coding RNAs, microRNA (miRNA), play a vital role in regulating biological and pathological processes leading to neurodegenerative diseases. Amyloid plaques, major hallmarks of AD, comprise abnormal aggregation of extracellular amyloid‐β peptides (Aβ) derived from Aβ precursor protein (APP). Neurofibrillary tangles consist of filamentous hyper‐phosphorylated tau proteins. Alpha‐synuclein (SNCA) plays a critical role in the pathogenesis of Parkinson’s and other synucleinopathies. Repressor Element 1‐Silencing Transcription (REST) factor is altered in ADRDs. We studied the role of miR‐153‐3p in AD risk and in regulating levels of critical proteins. miR‐153‐3p reduced APP, SNCA and

**Method:**

We measured miR153 levels in non‐cognitively impaired (NCI) and AD subject brain tissue samples from different recognized sources by qRT‐PCR as described (Wang et al). We utilized autopsy brain tissues and ADNI participants' genotyping and performed association studies of miR‐153‐3p and its single nucleotide polymorphisms (SNPs) with AD risk, and nine endophenotypes. We used iPSC‐derived neuronal cells, human cell lines and miRNA transfections to study the mechanism of miR‐153‐3p

**Result:**

Elevation of miR‐153‐3p is associated with a reduced probability of AD, while elevated REST associated with a greater likelihood of AD. MiR‐153 gene SNPs are associated with nine AD‐related endophenotypes. MiR‐153‐3p reduced REST, APP and SNCA 3’‐UTR activities and respective protein levels. MiR‐153‐3p treatment altered REST and neuronal differentiation in iPSC‐derived neuronal stem cells. RNA sequencing proteomics and interactome analysis revealed the role of miR‐153‐3p in axonal guidance.

**Conclusion:**

With the increased emphasis on comorbidities of AD and other neurodegenerative diseases, we identified that miR‐153‐3p, as a master regulator, reduced a key group of neurodegeneration‐related proteins. MiR‐153‐3p reduces APP, SNCA and REST expression, all pointing towards a therapeutic and biomarker potential in ADRDs. In addition to late‐onset cases, we will profile miR153 in brain tissue samples from early‐onset AD cases.

